# Exposure of BALB/c Mice to Diesel Engine Exhaust Origin Secondary Organic Aerosol (DE-SOA) during the Developmental Stages Impairs the Social Behavior in Adult Life of the Males

**DOI:** 10.3389/fnins.2015.00524

**Published:** 2016-01-25

**Authors:** Tin-Tin Win-Shwe, Chaw Kyi-Tha-Thu, Yadanar Moe, Yuji Fujitani, Shinji Tsukahara, Seishiro Hirano

**Affiliations:** ^1^Center for Environmental Health Sciences, National Institute for Environmental StudiesTsukuba, Japan; ^2^Division of Life Science, Graduate School of Science and Engineering, Saitama UniversitySaitama City, Japan; ^3^Center for Environmental Risk Research, National Institute for Environmental StudiesTsukuba, Japan

**Keywords:** diesel exhaust, secondary organic aerosol, brain, social behavior, mice

## Abstract

Secondary organic aerosol (SOA) is a component of particulate matter (PM) 2.5 and formed in the atmosphere by oxidation of volatile organic compounds. Recently, we have reported that inhalation exposure to diesel engine exhaust (DE) originated SOA (DE-SOA) affect novel object recognition ability and impair maternal behavior in adult mice. However, it is not clear whether early life exposure to SOA during the developmental stages affect social behavior in adult life or not. In the present study, to investigate the effects of early life exposure to DE-SOA during the gestational and lactation stages on the social behavior in the adult life, BALB/c mice were exposed to clean air (control), DE, DE-SOA and gas without any PM in the inhalation chambers from gestational day 14 to postnatal day 21 for 5 h a day and 5 days per week. Then adult mice were examined for changes in their social behavior at the age of 13 week by a sociability and social novelty preference, social interaction with a juvenile mouse and light-dark transition test, hypothalamic mRNA expression levels of social behavior-related genes, estrogen receptor-alpha and oxytocin receptor as well as of the oxidative stress marker gene, heme oxygenase (HO)-1 by real-time RT-PCR method. In addition, hypothalamic level of neuronal excitatory marker, glutamate was determined by ELISA method. We observed that sociability and social novelty preference as well as social interaction were remarkably impaired, expression levels of estrogen receptor-alpha, oxytocin receptor mRNAs were significantly decreased, expression levels of HO-1 mRNAs and glutamate levels were significantly increased in adult male mice exposed to DE-SOA compared to the control ones. Findings of this study indicate early life exposure of BALB/c mice to DE-SOA may affect their late-onset hypothalamic expression of social behavior related genes, trigger neurotoxicity and impair social behavior in the males.

## Introduction

Current epidemiological studies have indicated that inhalation of high levels of particulate matter (PM) is associated with damage to the central nervous system (Block and Calderón-Garcidueñas, 2009; Win-Shwe and Fujimaki, 2011; Block et al., 2012; Genc et al., 2012; Weisskopf et al., 2015). Ambient PM consists of primary particles emitted directly from sources, and secondary particles formed by photo-oxidation reactions of volatile organic compounds and gases in the atmosphere, which are known as secondary organic aerosols (SOAs) (Robinson et al., 2007). Diesel exhaust (DE) is a major component of PM and a major precursor of SOA (Kanakidou et al., 2005; Virtanen et al., 2012). Nowadays, the importance of SOA formation in urban areas is well-recognized, not only in the atmosphere but also in indoor environments (Wang et al., 2012; Youssefi and Waring, 2012). It has been reported that exposure to SOA emitted from coal-fired power plants may be associated with an increased risk of heart disease in susceptible animals (Wellenius et al., 2011). However, data showing the effects of SOA on central nervous system and neurobehavioral functions are very limited.

Human epidemiological studies and animal studies suggest that exposure to air pollution may lead to neurotoxicity (Costa et al., 2014). Recent review report indicates that the constituents of air fresheners can react with ozone to produce SOA and these pollutants adversely affect human health such as damage to the central nervous system and respiratory system and alteration of hormone secretion and immune responses (Kim et al., 2015). Previously, our research group has shown that the effects of primary particles such as carbon black nanoparticles and nanoparticle-rich diesel exhaust on brain inflammatory mediators, neurotransmitter system, memory function-related gene expression and learning performance in adult mice (Win-Shwe and Fujimaki, 2011). We have demonstrated that neuroinflammatory effects and neurotoxic effects of carbon black nanoparticle exposure by measuring inflammatory mediators and excitatory amino acid neurotransmitter levels in the hippocampus of BALB/c adult mice (Win-Shwe et al., 2006, 2008a). Furthermore, we have also shown that the effects of nanoparticle-rich diesel exhaust exposure on brain neurotransmitter, inflammatory biomarkers and learning ability in adult mice (Win-Shwe et al., 2008a,b, 2009, 2012a,b). We have generated SOA by adding ozone to diesel exhaust particles and established SOA inhalation chamber in our Research Institute. Using SOA inhalation chambers, we have shown that exposure to SOA for 3 months caused learning and memory impairment in adult male mice and SOA exposure for 1 month in female mice may cause changes in maternal behavior (Win-Shwe et al., 2014). Moreover, we have established the neonatal animal model for early detection of environmental pollutant-induced learning disability and reported that the diesel engine exhaust-derived secondary organic aerosol (DE-SOA) impairs olfactory-based spatial learning activity in preweaning mice (Win-Shwe et al., 2015). In that study, we have also shown that learning impairment was associated with modulation of N-methyl-D-aspartate (NMDA) receptor, signaling pathway gene CaMKII and inflammatory markers in the hippocampi of preweaning mice.

The purpose of the study was to investigate the early life exposure to DE-SOA during the gestational stages and lactation impairs the hypothalamic expression of social behavior-related genes and social behavior in adult life using a mouse model. We hypothesized that the potential toxic substances contained in DE-SOA may reach the brain via the olfactory nerve route or via the systemic circulation and cause social behavioral impairment in later life. Our study is the first report to show that exposure to DE-SOA during the developmental stage affects social performance and the related gene expressions in the hypothalamus of mature mice.

## Materials and methods

### Animals

Timed pregnant BALB/c mice (gestational day; GD 13) purchased from SLC Japan, Inc. (Tokyo, Japan) were exposed to clean air, diesel engine exhaust (DE), diesel engine exhaust origin secondary organic aerosol (DE-SOA) and gas only without diesel exhaust particles (Gas) from GD 14 to postnatal day (PND) 21 in the whole body exposure chambers. Food and water were given *ad libitum*. The day of the birth was recorded as PND 0 and the offspring were housed in cages with mothers under controlled environmental condition (temperature, 22 ± 0.5°C; humidity, 50 ± 5%; lights on 07:00–19:00 h). The pups were weaned at PND 21 and 5~6 pups of same sex were housed in a plastic cage. Social behavioral tests were started at approximately 13-week-old. Our social behavioral test consisted of sociability and social novelty preference task, social interaction with a juvenile mouse, and light-dark test. Behavioral testing was performed between 09:00 and 17:00 h. Before performing each test, the apparatus to be used was cleaned with 50% ethanol. After completing social behavioral test, these mice were sacrificed for brain sampling. The experimental protocols were approved by the Ethics Committee of the Animal Care and Experimentation Council of the National Institute for Environmental Studies (NIES), Japan.

### Generation of DE-SOA

DE-SOA was generated at the National Institute for Environmental Studies, Japan as described previously (Fujitani et al., 2009; Win-Shwe et al., 2014). An 81-diesel engine (J08C; Hino Motors Ltd., Hino, Japan) was used to generate diesel exhaust. The engine was operated under a steady-state condition for 5 h per day. In the present study, our driving condition of diesel engine was not simulated to any special condition as in the real world. The engine operating condition (2000 rpm engine speed and 0 Nm engine torque) in this study permits suppression of the generation of soot particles of relatively large size as well as the generation of high concentrations of nanoparticles. There are four chambers: a control chamber receiving clean air filtered through a HEPA filter and a charcoal filter (referred to as “clean air”), the diluted exhaust (DE which was without mixing O_3_), DE-SOA which was generated by mixing DE with ozone at 0.6 ppm after secondary dilution and gas without diesel exhaust particles. Secondary dilution ratio in DE and DE-SOA chambers were the same which resulted in the same particle and gaseous concentrations when O_3_ was not mixed. Actually, the concentrations of particles in DE-SOA was higher when O_3_ was mixed and concentrations of DE and DE-SOA were 113.19 ± 19.5 μg/m^3^ and 130.90 ± 31.2 μg/m^3^, respectively. The increased mass concentration was due to the generation of secondary particles. The temperature and relative humidity inside each chamber were adjusted to approximately 22 ± 0.5°C and 50 ± 5%, respectively. The particle characteristics were evaluated from the sample air taken from inside of the exposure chamber and presented in Table 1. In detail, sample air was taken from the breeding space of the inhalation chamber (2.25 m^3^) using stainless steel tubing. The gas concentrations (CO, CO_2_, NO, NO_2_, and SO_2_) were monitored using a gas analyzer (Horiba, Kyoto, Japan). CO and NOx concentrations in both chambers were similar, but NO and NO_2_ are different each other because NO was oxidized to NO_2_ by reacted with O_3_. The particle size distributions were measured using a scanning mobility particle sizer (SMPS 3034; TSI, MN). The sizes of the particles used in the present study were 25.42 ± 1.6 nm for DE and 28.30 ± 1.3 nm for DE-SOA. The particles were collected using a Teflon filter (FP-500; Sumitomo Electric, Osaka, Japan) and a Quartz fiber filter (2500 QAT-UP; Pall, Pine Bush, NY, USA), and the particle mass concentrations were measured using a Teflon filter. The particle weights were measured using an electrical microbalance (UMX 2, Mettler- Toledo, Columbus; OH, USA; readability 0.1 μg) in an air-conditioned chamber (CHAM-1000; Horiba) under constant temperature and relative humidity conditions (21.5°C, 35%). For the Quartz fiber filter, the quantities of elemental carbon (EC) and organic carbon (OC) were determined using a carbon analyzer (Desert Research Institute, NV, USA). EC to OC ratio in the present study were 0.14 ± 0.05 for the control chamber, 0.33 ± 0.02 for DE-SOA chamber and 0.32 ± 0.03 for DE exposure chamber. An analysis of the particle composition (DE and DE-SOA) showed that the percentage of OC relative to the total carbon in diluted exhaust was about 60% and the DE and DE-SOA was nearly same carbon composition.

**Table 1:** **Characteristics of diesel exhaust particles and gaseous compounds in the exposure chambers**.

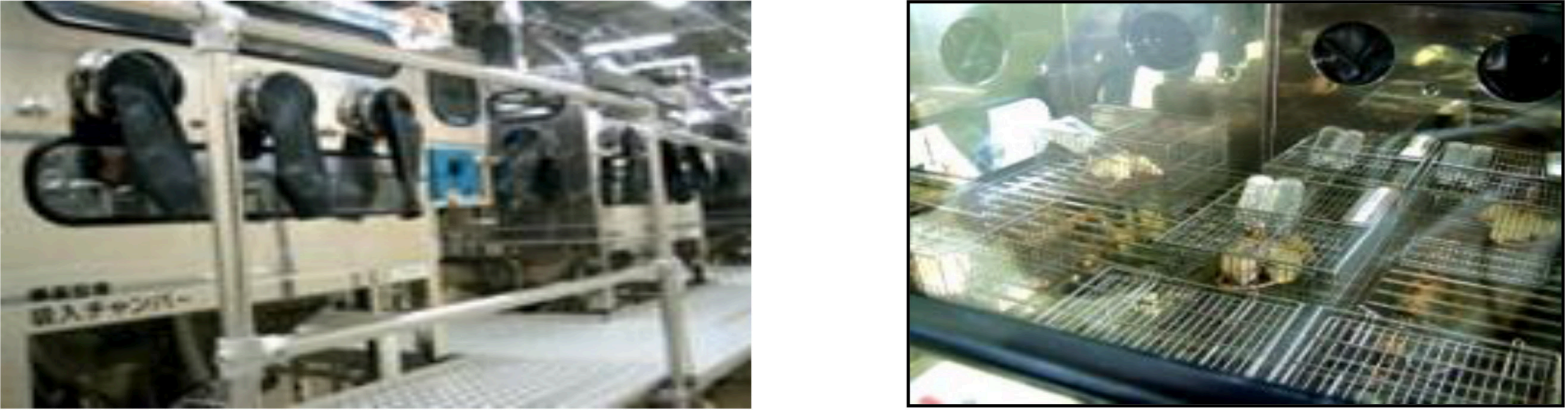							
	**Diesel exhaust particles**			**Temperature**	**Relative humidity**		
	**Size (nm)**	**Particle number (cm^−3^)**	**Concentration (μg/m^3^)**	**(°C)**	**(%)**	**EC/OC**	**WSOC/OC**
Clean air	–	0.87 ± 0.57	13.20 ± 2.78	23.58 ± 0.27	48.07 ± 0.77	0.14 ± 0.05	0.03 ± 0.04
DE-SOA	28.30 ± 1.28	2.74 × 10^6^ ±8.69 × 10^4^	130.90 ± 31.17	23.76 ± 0.19	48.40 ± 0.85	0.33 ± 0.02	0.11 ± 0.05
DE	25.42 ± 1.63	2.85 × 10^6^ ±6.10 × 10^4^	113.19 ± 19.46	22.97 ± 0.21	49.32 ± 0.87	0.32 ± 0.03	0.17 ± 0.11
Gas	–	5.55 ± 1.12	13.30 ± 1.45	23.67 ± 0.24	48.56 ± 1.08	0.14 ± 0.04	0.12 ± 0.06
**GASEOUS COMPOUNDS**							
	**CO (ppm)**	**SO_2_ (ppm)**	**NO_x_(ppm)**	**NO_2_ (ppm)**	**NO (ppm)**	**O_3_ (ppm)**	**CO_2_ (%)**
Clean air	0.21 ± 0.04	0.00 ± 0.00	0.00 ± 0.00	0.00 ± 0.00	0.00 ± 0.00	–	0.05 ± 0.00
DE-SOA	2.51 ± 0.07	0.00 ± 0.00	1.14 ± 0.03	0.99 ± 0.03	0.15 ± 0.03	0.07 ± 0.00	0.07 ± 0.00
DE	2.52 ± 0.07	0.01 ± 0.00	1.21 ± 0.03	0.43 ± 0.02	0.78 ± 0.02	–	0.07 ± 0.00
Gas	2.50 ± 0.06	0.00 ± 0.00	1.14 ± 0.03	0.99 ± 0.03	0.14 ± 0.03	0.06 ± 0.00	0.07 ± 0.00

Data were expressed as mean ± SD.

### Experimental schedule

Pregnant mice were exposed to clean air, DE, DE-SOA or gas in the whole-body exposure chamber (Shibata) for 5 h per day (from 22:00 h to 03:00 h) on 5 days of the week till PND 21. The male offspring mice at PND 21 were allocated into four different groups (*n* = 12 per group) as follows: (1) mice exposed to clean filtered air; (2) mice exposed to DE; (3) mice exposed to DE-SOA, and (4) mice exposed to gas only without diesel exhaust particles. On the day after the final exposure, the social performance of each mouse was examined using a three chamber sociability and social novelty preference performance, social interaction and light-dark test with Any-maze software video-assisted tracking system (Muromachi Kikai Co. Ltd., Japan).

### Behavioral tasks

All behavioral procedures were video-recorded, and data were analyzed by an experimental blind to the conditions.

#### Sociability and social novelty preference

The apparatus is a Plexiglas rectangular, three-chambered box (60 × 40 × 22 cm). The size of three chambers is equal. Dividing partition was made by clear Plexiglas, with small doorways (5 × 8 cm) allowing free access to each chamber. Small Plexiglas cups (diameter 8 cm; height, 10 cm) were used to house strangers and placed in each side chamber. Stranger mouse was placed on the left or right side. For habituation, subject mice from four different groups were first placed in center of the middle chamber and allowed to explore for 5 min. During habituation phase, each of two side chambers contained an empty Plexiglas cup. Following habituation, for the sociability test, a novel mouse (stranger 1, age-matched male) was enclosed in one of the cup and placed in one of the side chambers; the subject mice were allowed to explore for 10 min. The social novelty preference test was performed immediately after the sociability test. Another novel mouse (stranger 2, age-matched mouse) was enclosed in the other cup. And the subject mice were allowed to explore the two strangers for 10 min. The time spent in each Plexiglas cup was measured. The subject mouse was considered to be spent in cup when its head was facing the cup from a distance of within 3 cm.

#### Social interaction with a juvenile mouse

Male juvenile mice were used instead of adults to avoid any effect of mutual aggression (Moretti et al., 2005; Jung et al., 2013). A single male subject mouse was placed in a new cage which was identical to those in which the mice were normally housed and allowed to free for 10 min (habituation phase). Three to four week-old juvenile male mouse was introduced to the new cage and then allowed for 5 min (Test phase). Nose-to-nose sniffing, direct contact and close following (within < 1 cm) were recorded as social interaction parameters (Jung et al., 2013). The total time of social interaction with juvenile mouse was measured.

#### Light-dark test

The test apparatus consisted of a clear plastic box (40 × 20 × 25 cm) with a dark compartment (20 × 25 × 25 cm) and a light compartment (20 × 25 × 25 cm). The dark compartment had an open doorway (2 × 5 cm) that led to the light side of the apparatus, which was illuminated by a 40-W bulb (about 350 lux on the floor). Mice were moved from the living room to the testing room at least 1 h before the test. At the beginning of the tests, mice were removed from their cages, gently placed to the corner of the dark side of black box away from the doorway. Any-maze software were used to collect and store data. For each mouse, the following measurements were recorded for 10 min: total moving time, total time spent in the dark compartment, total time in the light compartment, number of transitions between the dark and light compartments, and the latency to the first emergence from the dark to the light compartment. Between the tests, the apparatus was thoroughly wiped to clean with 50% alcohol.

### Quantification of the expression levels of mRNAs

Twenty-four hours after the completion of the social behavioral tests, the mice from each group were sacrificed under deep pentobarbital anesthesia and the hypothalami were collected for mRNA analyses. Hypothalami samples were frozen quickly in liquid nitrogen then stored at –80°C until the total RNA was extracted. Briefly, total RNA extraction from the hypothalami samples was performed using the BioRobot EZ-1 and EZ-1 RNA tissue mini kits (Qiagen GmbH, Hilden, Germany). Then, the purity of the total RNA was examined, and the quantity was estimated using the ND-1000 NanoDrop RNA Assay protocol (NanoDrop, USA), as described previously (Win-Shwe et al., 2006, 2008a,b). Next, we performed first-strand cDNA synthesis from the total RNA using SuperScript RNase H^−^Reverse Transcriptase II (Invitrogen, Carlsbad, USA), according to the Manufacturer's protocol. Next, we examined the expression levels of 18S, estrogen receptor (ER) α and oxytocin receptor (OTR), cyclo-oxygenase (COX)-2, heme-oxygenase (HO)-1, interleukin (IL)1 β, tumor necrosis factor (TNF) α mRNAs by a quantitative real-time RT-PCR method using the Applied Biosystems (ABI) Prism 7000 Sequence Detection System (Applied Biosystems Inc., Foskr City, CA, USA). The tissue 18S rRNA level was used as an internal control. Primers (ER-α, NM_007956; OTR, NM_001081147, IL-1β NM_008361; COX2, NM_011198; HO1, NM_010442) were purchased from Qiagen, Sample & Assay Technologies. TNF-α primer (forward: 5′-GGTTCCTTTGTGGCACTTG-3′, reverse: 5′-TTCTCTTGGTGACCGGGAG-3′) was purchased from Hokkaido System Science (Hokkaido System Science, Hokkaido, Japan). Data were analyzed using the comparative threshold cycle method. Then, the relative expression levels of memory function-related genes and the related transduction pathway molecule mRNAs were individually normalized to the 18S rRNA content in the respective samples and expressed as mRNA signals per unit of 18S rRNA expression.

### Measurement of glutamate concentration

Glutamate concentration in the right hypothalamus of mice was measured using glutamate research ELISA assay kit (Ref: BA E-2300, Neuroscience. Inc., Tokyo, Japan) according to the manufacturer's instructions.

### Statistical analysis

All the data were expressed as the mean ± standard error (S.E.). The statistical analysis was performed using the StatMate II statistical analysis system for Microsoft Excel, Version 5.0 (Nankodo Inc., Tokyo, Japan). Paired *t* test was used to analyze the time approach to the empty cup and stranger 1, then stranger 1 and stranger 2. Messenger RNA data and glutamate concentration were analyzed by a one-way analysis of variance with a *post-hoc* analysis using the Bonferroni/Dunn method. Differences were considered significant at *P* < 0.05.

## Results

### Body and brain weight of adult mice exposed to clean air, DE, DE-SOA and gas during developmental period

To detect the general toxicity, body and brain weight were measured in adult male mice exposed to clean air, DE, DE-SOA and gas without particles at the time of sampling (Table 2). We did not find any significant changes between the control and exposure groups.

**Table 2:** **Body and brain weight of adult mice exposed to clean air, DE, DE-SOA, and gas during developmental period**.

**Exposure groups**	**Control**	**DE**	**DE-SOA**	**Gas**
Body weight (g)	28.22±0.39	28.99±0.97	28.03±0.57	29.07±0.53
Whole brain (mg)	462.11±5.19	469.75±5.14	468.67±4.57	472.33±4.90

### Effects of DE or DE-SOA on social behavior

#### Sociability

**Figure 1:**
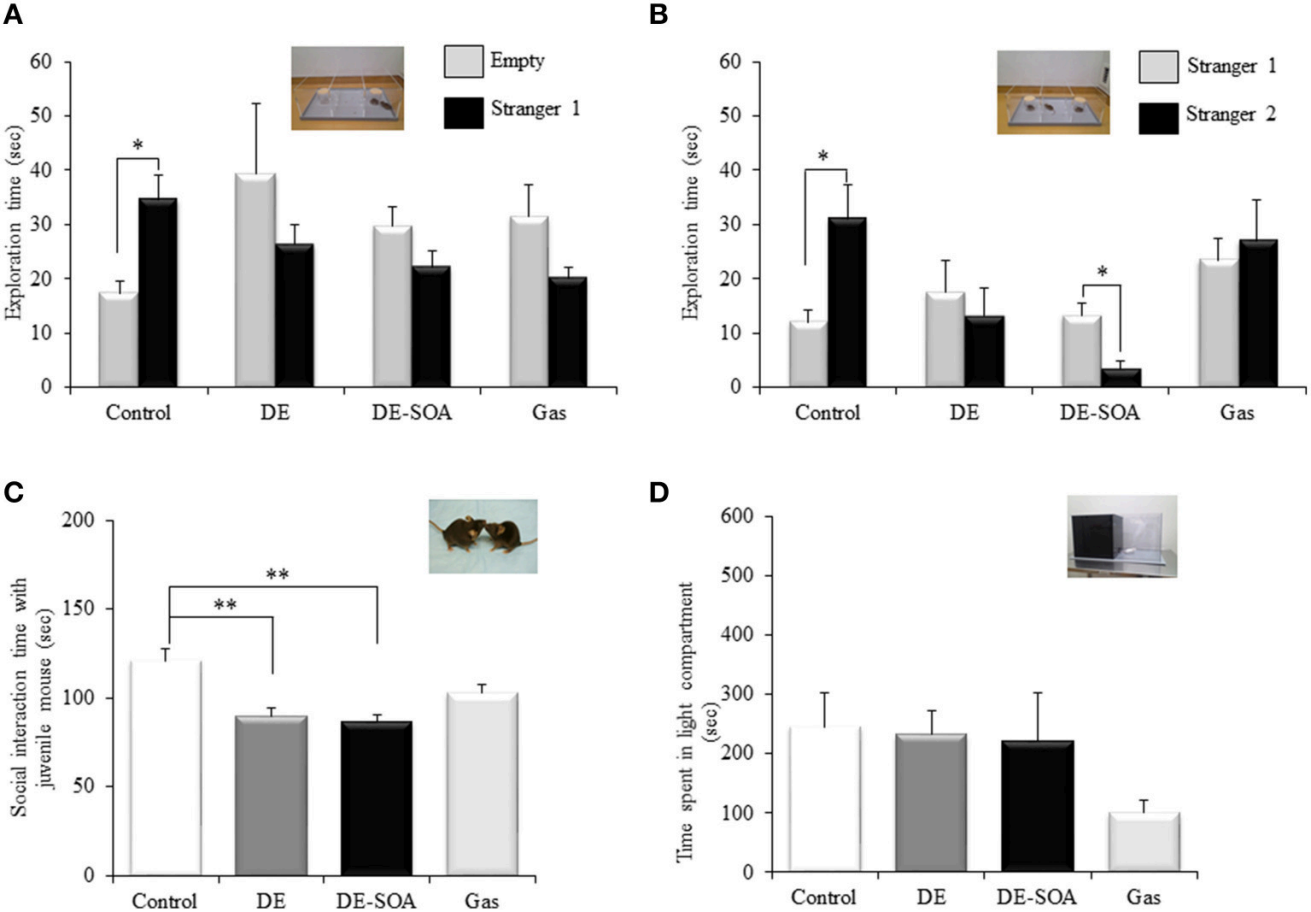
**Scores of the social behavioral tests. (A)** sociability test, **(B)** social novelty preference test, **(C)** social interaction test with juvenile mouse and **(D)** light dark test in 13 week-old male mice after developmental period exposure to clean air, DE, DE-SOA or gas without particles. Each bar represents the mean ± SE (*n* = 12, ^**^P < 0.01, ^*^P < 0.05).

The control mice spent more exploring time with the stranger 1 than the empty cup (Figure 1A, ^*^P < 0.05). In contrast, DE, DE-SOA, or gas exposed mice showed no preference for stranger 1, which could reflect decreased sociability.

#### Social novelty preference

The control mice spent more exploring time with the stranger 2 than the known mouse (stranger 1) (Figure 1B, ^*^P < 0.05). However, DE, DE-SOA or gas exposed mice showed no preference for novel one (stranger 2). DE-SOA exposed mice preferred old one (stranger 1) significantly compared to the novel one (stranger 2) (Figure 1B, ^*^P < 0.05), which may indicate that they may have poor social novelty preference.

#### Social interaction with a juvenile mouse

Nose-to-nose sniffing, direct contact and close following (within < 1 cm) were recorded as social interaction parameters. The total time of social interaction with juvenile mouse was measured. We found that DE or DE-SOA exposed mice showed significantly decreased interaction time with juvenile mouse compared to the control mice (Figure 1C, ^*^P < 0.05).

#### Light-dark test

Light-dark box is a characteristic tool used in the assessment of anxiety. In the present study, total moving time, total time spent in the dark compartment, total time in the light compartment, number of transitions between the dark and light compartments, and the latency to the first emergence from the dark to the light compartment. However, we did not find any significant difference between the control and exposure groups. Time spent in the light compartment was shown in Figure 1D.

### Effects of DE or DE-SOA on the hypothalamic expression of social behavior-related genes

**Figure 2:**
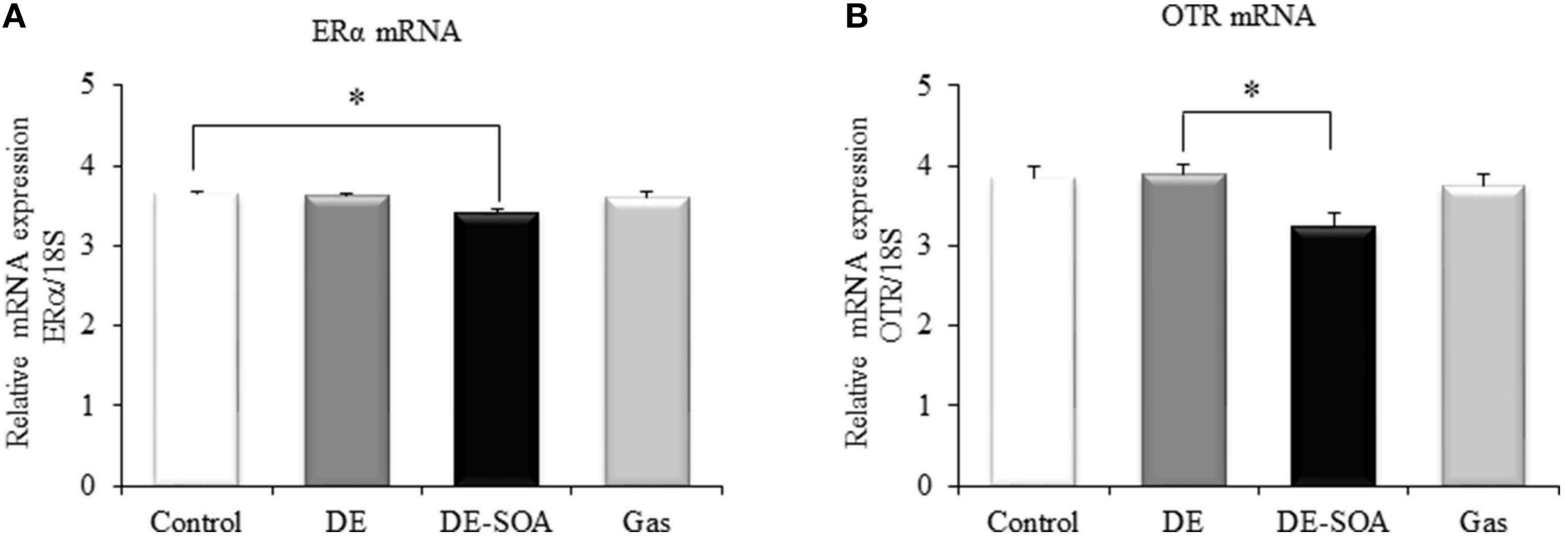
**Hypothalamic expression of social behavior-related genes**. Messenger RNA expression level of **(A)** ERα and **(B)** OTR in the hypothalami of 13 week-old male mice after developmental period exposure to clean air, DE, DE-SOA or gas without particles. Each bar represents the mean ± SE (*n* = 12, ^*^P < 0.05).

Recently, Ervin and colleagues have demonstrated that estrogens are involved in various social behavior such as social preferences, aggression and dominance, and learning and memory (Ervin et al., 2015). Moreover, it was reported that the ERα in the medial amygdala and ventromedial nucleus of the hypothalamus palys a role in social recognition, anxiety and aggression (Spiteri et al., 2010). Our present study has shown that expression of ERα was decreased significantly in DE-SOA exposed group compared to the control group (Figure 2A, ^*^P < 0.05).

The role of the OT in the normal processing of socially relevant clues has been extensively investigated in genetically-modified rodent models. Social deficit has been observed in OT receptor knockout mice (Winslow and Insel, 2002; Crawley et al., 2007). In the present study, expression level of OTR in the hypothalamus was examined and found that OTR mRNA was decreased significantly in mouse exposed to DE-SOA compared to DE group (Figure 2B, ^*^P < 0.05) and tended to decrease compared to the control group.

### Effects of DE or DE-SOA on the hypothalamic expression of inflammatory and oxidative stress marker genes

**Figure 3:**
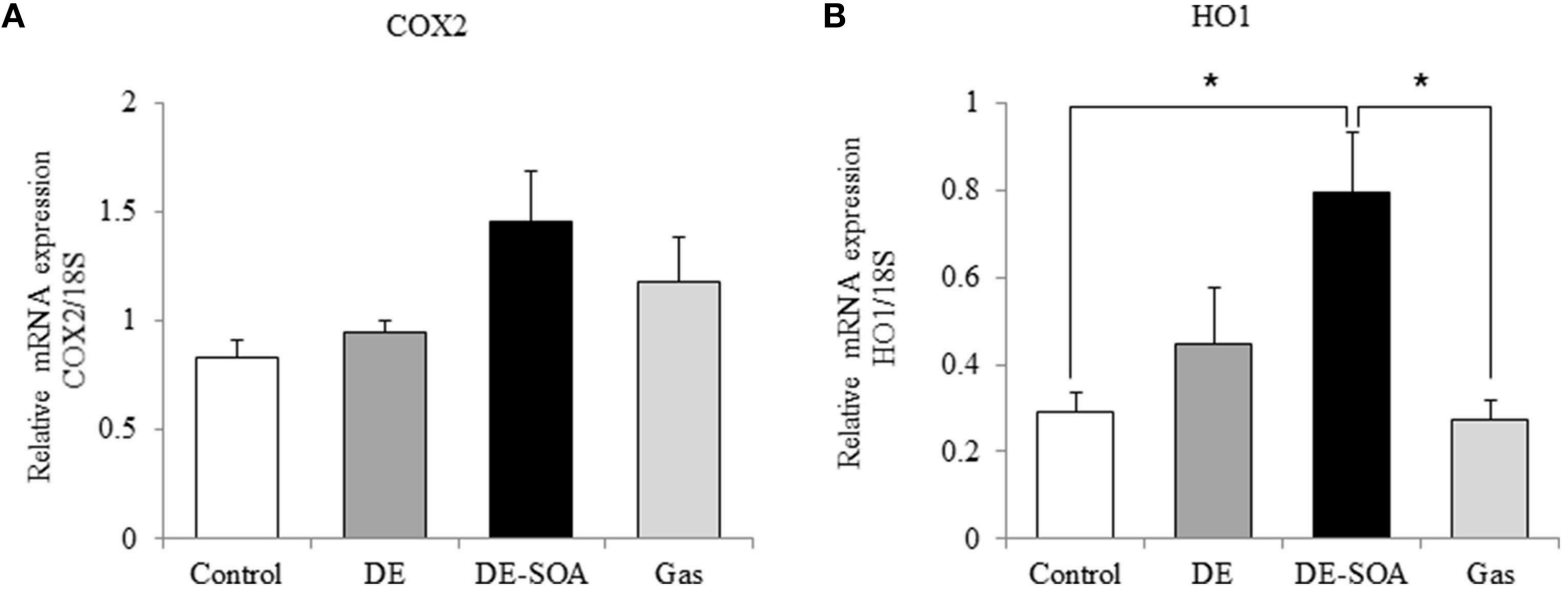
**Hypothalamic expression of inflammatory and oxidative stress markers**. **(A)** COX2 and **(B)** HO1 mRNAs in 13 week-old male mice after developmental period exposure to clean air, DE, DE-SOA or gas without particles. Each bar represents the mean ± SE (*n* = 12, ^*^P < 0.05).

To detect the inflammatory response in the brain, we investigated the expression level of potent inflammatory cytokines such as IL-1 β, TNF-α and potent inflammatory marker COX2. The expression levels of IL-1 β and TNF-α were not different between the control and the exposure groups (data not shown). COX is the enzyme responsible for the conversion of arachidonic acid to prostaglandin, which is involved in the inflammatory response. COX2 is an inducible form and is released at the site of inflammation. In the present study, COX2 mRNA was tended to increase in the DE-SOA-exposed group compared with the control ones (Figure 3A). To understand the mechanism underlying the inflammatory response in the hypothalamus of mice exposed to DE-SOA, we also examined the expression of the oxidative stress marker HO1 and found that HO1 mRNA was significantly upregulated in the DE-SOA-exposed group compared with the control group (Figure 3B, ^*^P < 0.05).

### Effects of DE or DE-SOA on the hypothalamic level of neuronal excitatory marker

**Figure 4:**
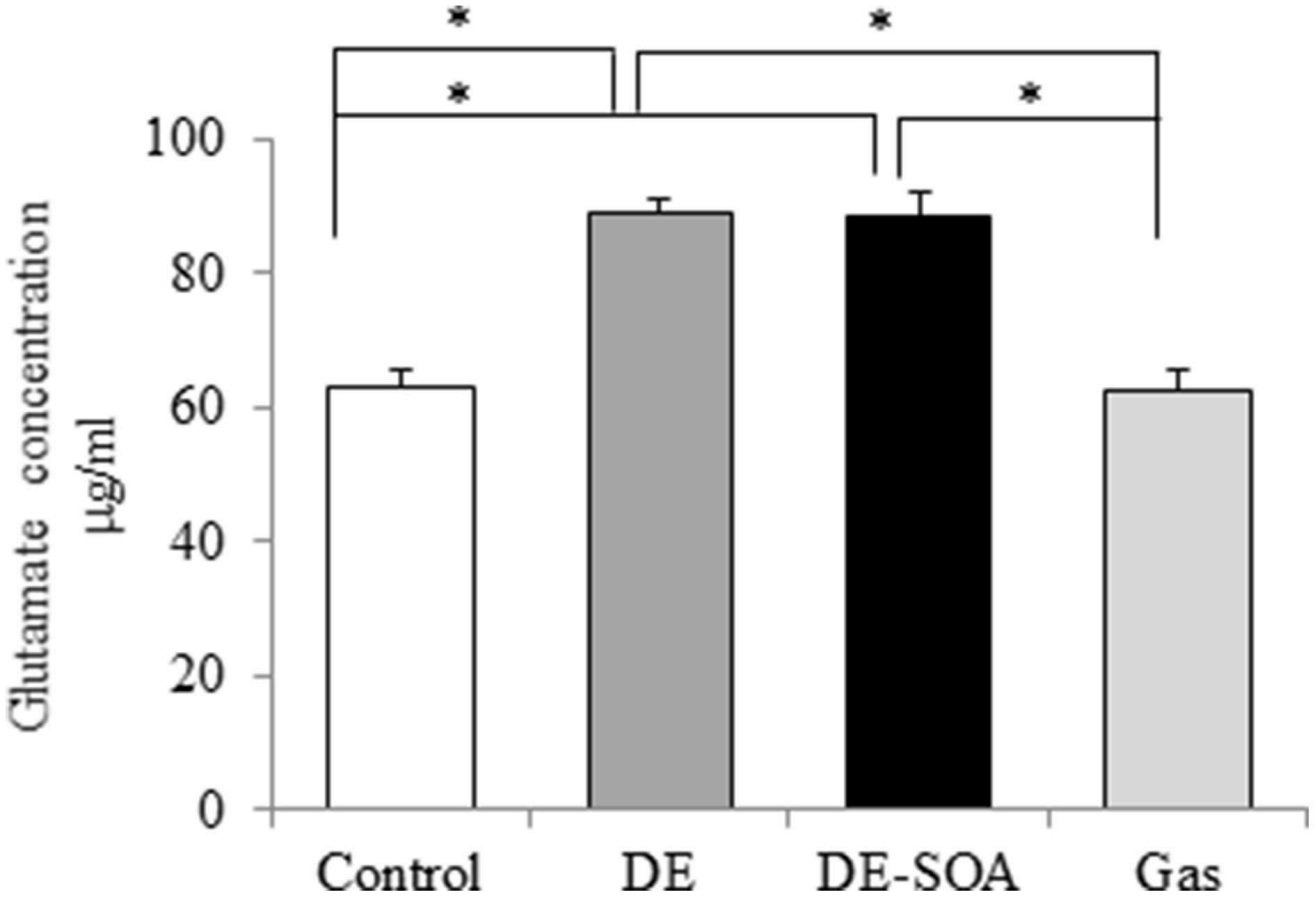
**Hypothalamic levels of neuronal excitatory marker**. Glutamate concentration in the hypothalami of 13 week-old male mice after developmental period exposure to clean air, DE, DE-SOA or gas without particles. Each bar represents the mean ± SE (*n* = 12, ^*^P < 0.05).

Glutamate is the major excitatory neurotransmitter in the mammalian central nervous system (Fonnum, 1984) and the excessive increase in extracellular glutamate level, known as excitotoxicity, triggers the death of neurons (Choi and Rothman, 1990). We detected glutamate concentration in the mouse hypothalamus and found that glutamate secretion was increased remarkably in mice exposed to DE or DE-SOA during fetal and neonatal developmental period (Figure 4, ^**^P < 0.01).

## Discussion

The major findings in the present study indicate that exposure to DE-SOA during brain developmental period may impair some social behaviors in adult male BALB/c mice accompanied with modulation of expression of ERα and OTR, inflammatory mediator COX2 and oxidative stress marker HO1 in the hypothalamus. We suggest that, although the potential toxic substances contained in DE-SOA have not yet been identified, they may reach the brain via placenta of pregnant mice during fetal period and via the olfactory nerve route or systemic circulation during neonatal period and induce neurotoxicity. These effects may be persistent because we exposed the mice during early brain developmental period and investigated the social behavior and related gene expression in later age.

Previous epidemiological evidences have shown the existence of positive associations between the inhalation of elevated levels of PM and pulmonary and cardiovascular morbidities and mortality in susceptible populations (Dockery, 1993; Peters et al., 1997; Penttinen et al., 2001; Pope et al., 2002). Recent reports indicate that the central nervous system is an important target for air pollution to cause adverse health effects such as neurodevelopmental and neurodegenerative disorders like autism spectrum disorder (ASD) and Alzheimer's disease (Calderón-Garcidueñas et al., 2004; Deth et al., 2008; Lee et al., 2010; Landrigan et al., 2012). DE is a major source of ambient PM and one of the major precursors for SOA formation. SOAs are formed in the atmosphere by oxidation of products originating from anthropogenic and biogenic volatile organic compounds (Virtanen et al., 2012). SOA formation may occur not only in the atmosphere, but also in indoor environments where laser printer, copiers were used (Wang et al., 2012; Youssefi and Waring, 2012).

First, we have shown that a single intranasal administration of SOA induces inflammatory responses in the lungs by modulating the expressions of proinflammatory cytokines, transcription factors, and inflammation-responsive neurotrophins (Win-Shwe et al., 2013). Currently, we have generated SOA by adding ozone to diesel exhaust particles and establish SOA inhalation chamber in our Research Institute. Using SOA inhalation chambers, we have shown that exposure to SOA for 3 months caused learning and memory impairment in adult male mice and SOA exposure for 1 month in female mice may cause changes in maternal behavior (Win-Shwe et al., 2014). Recently, we have established the neonatal animal model for early detection of environmental pollutant-induced learning disability and reported that DE-SOA impairs olfactory-based spatial learning activity in preweaning mice (Win-Shwe et al., 2015). In that study, we have also shown that learning impairment was associated with modulation of NMDA receptor, signaling pathway gene CaMKII and inflammatory markers in the hippocampus. From these findings, we suggest that glutamate, a ligand for NMDA receptor, may involve SOA induced neurobehavioral dysfunctions.

Human studies have reported that children from Mexico City with prefrontal lesions exposed to air pollution showed cognitive deficits (Calderón-Garcidueñas et al., 2008a, 2011). An association has also been shown between air pollution and cognitive impairment in healthy individuals, including adult and elderly women (Calderón-Garcidueñas et al., 2004, 2008b). An *in vitro* study indicated that decreased phagocytic activity was found in human macrophages exposed to SOA from alpha-pinene, and IL-8 production was increased in pig explants exposed to SOA from 1,3,5-trimethlbenzene with high particle numbers (Gaschen et al., 2010). However, it is not clear whether an association may exist between exposure to SOA derived from DE and higher functions of the brain such as social behavior.

In the present study, the control group only approached longer time to stranger 1 cup compared to empty cup in sociability test. In social novelty preference test, the control group approached longer time to stranger 2 cup compared to stranger 1 cup. These findings suggests that treatment groups such as DE, DE-SOA or gas without particles groups may have poor communication with new partner. We have also examined social behavioral related genes such as ERα and OTR and proinflammatory cytokines and potent inflammatory marker and oxidative stress marker and found that the expression level of COX2 and HO1 mRNA were increased in the hypothalamus of DE-SOA exposed mice. Components of DE-SOA may exert their deleterious effects directly on the central nervous system, the possibility and the extent of a peripheral contribution to the central effects should be considered. It was reported that high levels of circulating proinflammatory cytokines may negatively affect the he central nervous system (Block and Calderón-Garcidueñas, 2009; Calderón-Garcidueñas et al., 2013), and the blood-brain barrier may represent an important site for air pollution induced neurotoxicity.

To detect the possible mechanism of the action of SOA in social impairment, we have examined glutamate concentration in hypothalamus. Glutamate is one of excitatory amino acid neurotransmitters. Neurotransmitters play many critical roles in the neuronal transmission and maintenance of many higher brain functions. Deviation of neurotransmitter from the normal physiological level may lead to certain malfunctions and pathological states of the brain. Under normal conditions, extracellular glutamate is maintained at safe physiological concentrations by a number of buffering mechanisms which include uptake of glutamate by glial cells and its conversion by glutamine synthetase or glutamate decarboxylase to the nontoxic glutamine (Bezzi et al., 1999; Rauen et al., 1999). In the present study, glutamate concentration was remarkably increased in DE or DE-SOA exposed mice. It is suggested that there may be association exists between increased glutamate neurotransmission and impaired social behavior. Amino acid transporters present in both the neurons and the glial cells are critically important for the normal function of glutamatergic transmission, as well as for the maintenance of extracellular glutamate levels below potentially excitotoxic concentrations (Kanai et al., 1993, 1995). The possible reasons for increased glutamate level in DE-SOA exposed mice are due to blockade of re-uptake by glutamate transporter in the presynaptic neurons and decreased downstream enzymes such as glutamic acid decarboxylase (GAD) 67 and GAD 65 for gamma amino butyric acid (GABA) synthesis. Further studies are needed to evaluate the role of glutamate transporters in SOA induced impaired behavior.

Recent report has indicated that estrogens are involved in various social behavior such as social preferences, aggression and dominance, and learning and memory (Ervin et al., 2015). It was also reported that the ERα in the medial amygdala and ventromedial nucleus of the hypothalamus palys a role in social recognition, anxiety and aggression. (Spiteri et al., 2010). Our present study has shown that expression of ERα was decreased in DE-SOA exposed group compared to the control group. Moreover, impaired social recognition has been observed in OT peptide and receptor knockout mice (Winslow and Insel, 2002; Takayanagi et al., 2005). Oxytocin can reduce repetitive behavior in subjects with autism (File et al., 1998) and promote social behavior in high functioning ASDs (Andari et al., 2010). Recent reports have indicated that intranasal oxytocin reduces psychotic symptoms and improves theory of mind and social performance in schizophrenia patients (Pedersen et al., 2011; Davis et al., 2013). In the present study, although statistically not significant, OTR mRNA expression tends to decrease in DE-SOA exposed mice compared to the other groups. Taken together, ERα and OTR, at least in part, may play a role in DE-SOA induced social behavioral disturbance.

Our present results indicate that brain developmental period exposure to diesel exhaust origin SOA may impair some social behavior in adult BALB/c mice. In the present study, not only the offspring, but also the dam were exposed to DE-SOA during gestational and lactational periods. Normal maternal behaviors such as nesting, licking, crouching and retrieving were observed during exposure period. Therefore, impairment of social behavior in adult male mice might not due to abnormal maternal caring during brain developing periods. Glutamate neurotransmission and ERα-OTR signaling pathway in the hypothalamus may take part in DE-SOA induced social behavioral impairment. Further studies are needed to explore the effects of DE-SOA on other brain targets such as amygdala and hippocampus and other social behavior such as sexual, aggressive or anxiety after early life exposure. Furthermore, animal experiments showed the same pattern of neurotoxic effects such as increased oxidative stress markers, increased neuroinflammatory mediators and age factor, as in humans, suggesting that animal studies would be useful predictors of human outcomes (Costa et al., 2014). Finally, *in vitro* studies are needed for better understanding of air pollution induced neurotoxicity and their consequences.

## Author contributions

TW and SH designed this research; YF, arranged the exposure system; CK and YM performed the behavioral tests and molecular analyses; TW wrote the article; SH and ST critically revised the article.

### Conflict of interest statement

The authors declare that the research was conducted in the absence of any commercial or financial relationships that could be construed as a potential conflict of interest.
